# Positive changes in daily life? A meta‐analysis of positive psychological ecological momentary interventions

**DOI:** 10.1111/aphw.70006

**Published:** 2025-02-10

**Authors:** Samuel Tomczyk, Christina Ewert

**Affiliations:** ^1^ Department Health and Prevention, Institute of Psychology University of Greifswald Greifswald Germany; ^2^ German Center for Child and Adolescent Health (DZKJ), partner site Greifswald/Rostock Greifswald Germany; ^3^ Department of Psychology University of Potsdam Potsdam Germany

**Keywords:** ecological momentary assessment, eHealth, mobile health, positive psychology, SDG 3: Good health and well‐being, well‐being

## Abstract

Positive psychological interventions (PPI) hold promise for boosting well‐being and quality of life in diverse populations, but not much is known about their efficacy as ecological momentary interventions (EMIs, e.g. via mobile applications) in daily life. This meta‐analysis uses random‐effects models to examine the efficacy of PPI‐EMIs compared to control groups (active or passive) and exploring study region, age, gender, and risk of bias as moderators. Overall, 16 studies were included (*N* = 3397, 69.1% female, *M*_age_ = 21.87, *SD* = 13.02). We observed clinically significant effects in favor of the intervention for positive affect at posttest (*k* = 6; *g* = 0.29; *p* = 0.05) and well‐being at follow‐up (*k* = 5; *g* = 0.21; *p* = 0.13). No significant moderator effects were found. The number of studies was small for each outcome, risk of bias was mixed, and heterogeneity of effects was moderate to high for most outcomes.

## INTRODUCTION

Positive psychological interventions (PPIs) aim to foster well‐being, positive cognitions, experiences, and emotions (Keyes et al., 2012; Lyubomirsky & Layous, 2013; Seligman, 1999). They have received increased attention in the past 20 years, as a counterpoint to psychopathology, namely, a focus on what makes (and keeps) people happy and resilient instead of what makes (and keeps) them ill. Recently, with global crises like climate emergencies, the COVID‐19 pandemic, and ongoing wars (e.g. in Ukraine), Waters et al. (2022) discussed PPIs as a means of strengthening mental health during crisis. Accordingly, PPIs can have buffering (i.e. diminishing negative mental health), bolstering (i.e. maintaining positive mental health despite challenging circumstances), and building (i.e. transforming oneself by gaining enhanced meaning) effects on mental health. Indeed, several studies found that PPIs were able to reduce negative affect and increase positive affect and resilience during the COVID‐19 pandemic (e.g. Brouzos et al., 2023; García‐Álvarez et al., 2022; Tomczyk et al., 2023).

While there are many different conceptualizations of PPIs, this study defines PPIs as psychological interventions (e.g. trainings, exercises, or therapies) that aim to strengthen positive thoughts, emotions, and behaviors to achieve positive, sustainable effects on well‐being (Bolier et al., 2013; Carr et al., 2021; Ciarrochi et al., 2021; Sin & Lyubomirsky, 2009). Furthermore, PPIs should be grounded in positive psychological theory (Parks & Layous, 2016), such as broaden‐and‐build (Fredrickson, 2004), and provide empirical support for their effectiveness. Thus, PPIs are conceptualized in contrast to clinical interventions that primarily focus on reducing (transdiagnostic) illness symptoms or treating specific disorders. Research shows that PPIs that focus on positive experiences and activities (e.g. three good things, gratitude letters) can lead to a spiral of positive emotions (Fredrickson, 2004; Lyubomirsky & Layous, 2013), since they increase the mental availability of positive memories, behavioral options, and emotional reactions as opposed to more negative ones. In a large review of 347 primary studies, Carr et al. (2021) found small to moderate effects of PPIs on well‐being, quality of life, depression, anxiety, and stress that persisted over a 3‐month follow‐up period. However, the studies varied in their methodology and largely investigated small and selective samples; hence, a reanalysis of highly cited meta‐analyses of PPIs (White et al., 2019) reported smaller but still significant PPI effects on well‐being when considering such methodological differences. Overall, there are relevant effects of PPIs on facets of well‐being, yet their impact in daily life is not very well‐studied.

To address this issue, ecological momentary interventions (EMIs) are promising interventions in daily life that provide prompts in natural settings and in real life (as opposed to laboratory settings) (Balaskas et al., 2021; Heron & Smyth, 2010; Parmar & Sharma, 2017). They are based on ecological momentary assessment (EMA), also referred to as ambulatory assessment or experience sampling (e.g. daily diaries) that is meant to capture fluctuations across situations and events in participants' biobehavioral processes, thoughts, feelings, and behaviors (Fahrenberg et al., 2007; Kubiak & Stone, 2012; LaCaille et al., 2013; Reininghaus et al., 2016; Shiffman et al., 2008; Wrzus & Neubauer, 2023). EMA allows for psychological research on experiences and states in daily life, and consequently, EMIs are just‐in‐time interventions that are connected to specific EMA prompts or reports (e.g. cue sensitivity in smoking cessation interventions) (Rodgers et al., 2005; Shiffman et al., 2008). Therefore, EMIs can be personalized or customized (e.g. interventions based on specific EMA signals), have high ecological validity (due to their implementation in daily life), and can consider specific contexts or situations (e.g. promoting tailored breathing techniques during high‐stress situations like exams). A few studies on PPIs as EMIs (e.g. gratitude and mindfulness) show positive effects of these interventions on outcomes (e.g. well‐being) (Runyan & Steinke, 2015; Shim et al., 2022; Zainal & Newman, 2023), but the evidence has not been rigorously reviewed yet.

### Research questions

To address these questions, this meta‐analysis aims to synthesize effects of previous positive psychological EMIs on key outcomes (well‐being, quality of life, and positive and negative affect) that were significantly influenced by traditional PPIs (Carr et al., 2021; Duan et al., 2022; Lim & Tierney, 2023). In addition, this study considers potential moderators of effectiveness, namely, age, gender, study region, and risk of bias, to increase comparability with previous meta‐analyses and reviews of either PPIs or EMA/EMI studies (Balaskas et al., 2021; Carr et al., 2021; Wrzus & Neubauer, 2023).

## MATERIALS AND METHODS

This meta‐analysis followed recommendations by the Cochrane Collaboration (Higgins et al., 2019) and the PRISMA statement (Page et al., 2021), and it was preregistered in April 2024 (PROSPERO: CRD42024528187) via PROSPERO (Schiavo, 2019). Both authors and student assistants conducted the research (see Acknowledgments). Additional material (e.g. funnel plots) can be found in the online Supporting Information.

### Procedure

The literature search was performed from January to May 2024 in three scientific data bases (PubMed, APA PsycINFO/EBSCOhost, and Web of Science). In addition, relevant scientific and professional associations in the field of positive psychology and EMA/EMI research (Society for Ambulatory Assessment (SAA), German Psychological Association (DGPs), Deutsche Gesellschaft für Positive Psychologie (DGPP), European Network for Positive Psychology (ENPP), Western Positive Psychology Association (WPPA), International Positive Psychology Association (IPPA), and Deutschsprachiger Dachverband für Positive Psychologie e.V. (DACH‐PP)) were contacted to identify relevant gray and unpublished literature. Search terms for each data base (see Appendix A1) were based on “ecological momentary interventions” and “positive psychological interventions” and combinations thereof, using Boolean operators (OR, AND).

### Eligibility criteria

The inclusion and exclusion criteria were as follows: The search was limited to controlled studies (with active or passive control groups) in German or English, published between 2005 and 2024 (since the term EMI was coined in 2005 by Patrick et al. (2005)). There were no limitations regarding study population, but studies should provide quantitative data to measure the effect of PPIs delivered as EMIs (i.e. at least one assessment before and after the intervention for an analysis of prepost effects). Hence, studies without sufficient data (e.g. qualitative studies, reviews, or case studies), EMA/EMI design or a PPI (as defined above) were excluded. If studies did not report sufficient quantitative data for the meta‐analysis, but all other criteria were fulfilled, study authors were contacted three times to gain additional data. Studies were excluded if relevant data could not be obtained.

### Screening

**FIGURE 1:**
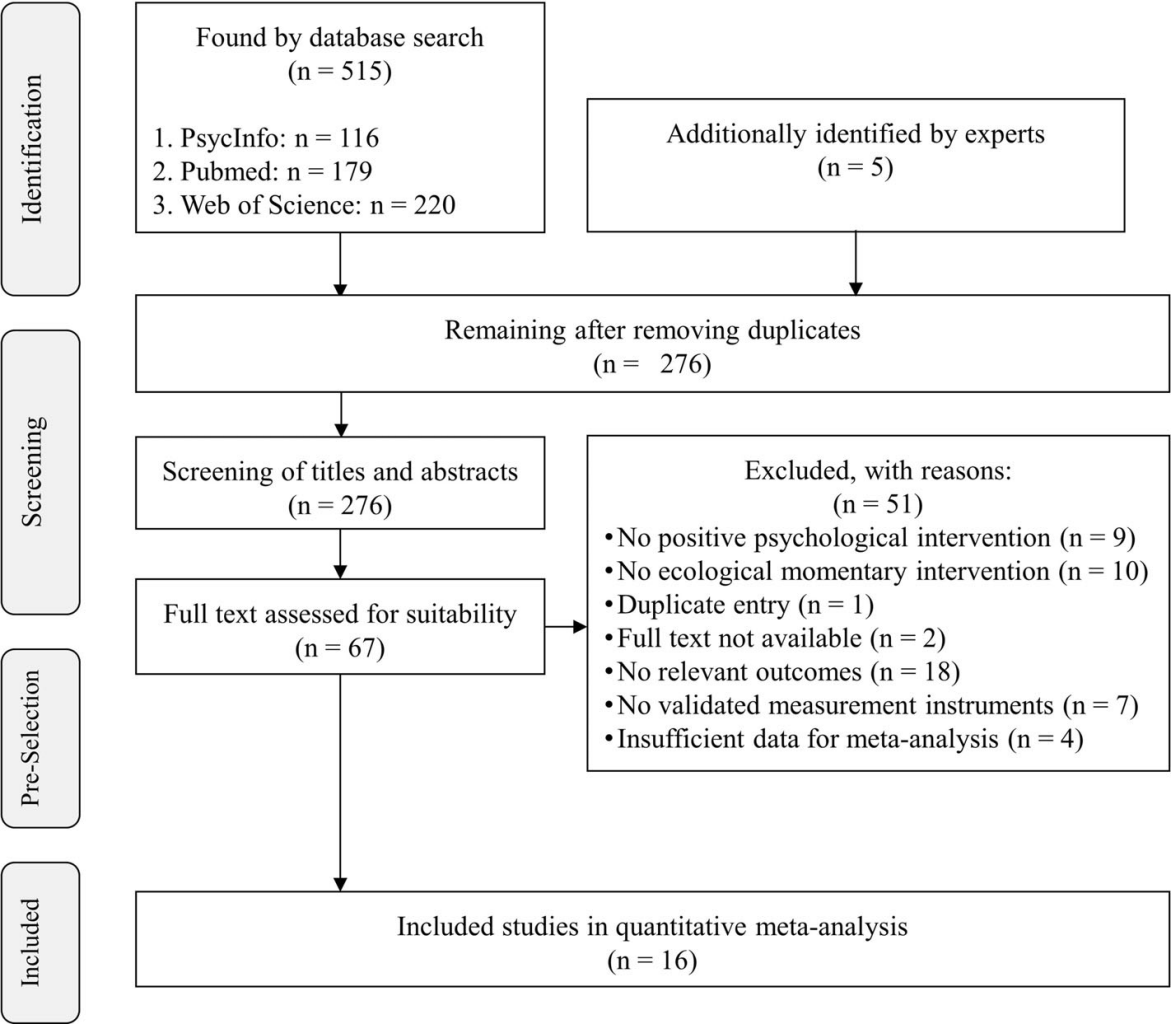
PRISMA flowchart of the study selection procedure*.*

Following the data base search, results were exported to the reference management software Zotero to identify duplicates and initiate the screening of titles and abstracts, which was supported by two student assistants (see Acknowledgments). Both of them screened the results independently, and discussed differences to achieve a consensus on relevant full texts. For two steps, (1) screening of titles and abstracts as well as (2) screening of full texts, Cohen's kappa was calculated to reflect interrater agreement (Rubin et al., 1985). Full text screenings resulted in a final sample of *k* = 16 studies for the quantitative meta‐analysis (see Figure 1 for the PRISMA flowchart).

### Data extraction

An Excel 2018 file was created for data extraction. Data comprised authors, year and title of the publication/study, sample size (for intervention and control groups), age (mean and standard deviation), gender (relative frequency of female participants), study region (coded as 1 = USA/Canada, 2 = Europe, 3 = Asia), and a brief description of the intervention. A second table contains means and standard deviations of relevant outcomes (assessed at pre‐ and posttest, and follow‐up, if available) for intervention and control groups as well as the utilized outcome measures. During data extraction, we checked for similar groups of authors (Wrzus & Neubauer, 2023) to avoid repeated analysis of the same data set.

### Risk of bias assessment

Risk of bias within and between studies was assessed as an indicator of study quality with the revised Cochrane risk‐of‐bias tool for randomized trials (RoB2) (Minozzi et al., 2022). The tool was completed by two experienced raters of studies in systematic reviews and meta‐analyses and assesses aspects of trial design, conduct, and reporting. Following an initial assessment of interrater agreement (Cohen's kappa), a consensus decision of remaining differences was made by discussing independent ratings, leading to summary judgments of low or high risk of bias or some concerns. This information was reported in the overview of studies and included in the meta‐analysis as a potential moderator.

### Data analysis

For the analysis, we included the outcomes well‐being, quality of life, and positive or negative affect that were assessed in multiple studies (and thus usable for meta‐analysis) (Versluis et al., 2016), based on positive psychological theory, and using validated, psychometrically tested instruments (a list of these measures is provided in Table 2). For each study, group means of intervention and control groups were used to calculate standardized mean differences as effect sizes. Subscales were summarized into global scores to allow for single outcome effect sizes in each study (Al‐Refae et al., 2021; Mirabito & Verhaeghen, 2023; Rocamora González et al., 2022). Time points were coded as pre‐intervention (T0), post‐intervention (T1), and follow‐up (T2), if available. If any relevant data were missing (e.g. means, standard errors, and group sizes), primary study authors were contacted (up to three times) to provide additional information.

The analysis was done in three steps, separately for each outcome, in accordance with previous meta‐analyses (Bolier et al., 2013; Carr et al., 2021; Chakhssi et al., 2018; Koydemir et al., 2021): First, standardized mean differences of T0 and T1 were compared (using pooled standard deviations). Second, effect size differences between intervention and control group were calculated (∆Cohen's d), and third, Hedge's g was calculated as an overall effect size (Higgins et al., 2019). In accordance with previous research (Ewert et al., 2021), if studies reported a follow‐up assessment, and at least three studies per outcome were available, an additional test of effect sizes between T1 and T2 was calculated. Due to the novelty of the field and the anticipated small number of specific publications, we chose to perform meta‐analysis with at least three studies (as opposed to 5–10 studies, as recommended by some scholars (Borenstein et al., 2021)). In line with recommendations, effects were classified as small (0.2 ≤ *g* < 0.5), moderate (0.5 ≤ *g* < 0.8), and large (0.8 ≤ *g*) (Cohen, 1988).

In addition, we performed tests of heterogeneity, sensitivity, and influential outliers as well as subgroup analyses (for region and risk of bias) and meta‐regression models (for age and gender), if at least three studies were available per outcome (Ewert et al., 2021; Higgins et al., 2003). In these cases, a value of *p* < .05 and an alpha error of .05 were assumed. We utilized R Studio, version 2023.9.1.494, and the packages metafor (Viechtbauer & Cheung, 2010) and dmetar (Harrer et al., 2022) for the analysis. To guarantee independence of effect sizes (Schewe et al., 2014), we selected the measurement instrument for each study that presented the best conceptual fit for the construct in question (e.g. well‐being). If one study reported multiple intervention groups, weighted mean effects were calculated to maintain independent effects (Borenstein et al., 2021; Higgins et al., 2019). A random‐effects estimation model was chosen since we assumed heterogeneity between studies, due to differences in outcomes, measurement instruments, and target groups (Borenstein et al., 2021; Lipsey & Wilson, 2001). Heterogeneity between studies was tested using restricted maximum likelihood estimation, as recommended for continuous outcomes (Veroniki et al., 2015). The confidence interval was calculated using the Knapp‐Hartung method (Knapp & Hartung, 2003; Langan et al., 2019). Cochran's Q and Higgins' and Thompson's *I* ^2^ (Higgins et al., 2003) were reported as indicators of small (25%), moderate (50%), or high (75%) heterogeneity (Higgins & Thompson, 2002). In addition, *τ* ^*2*^ was reported as an indicator of overall heterogeneity (a value close to 0 indicates low heterogeneity across studies) (Quintana, 2015).

To identify influential outliers, we created Baujat plots (Baujat et al., 2002) for visual inspection and supported the analysis using the influence function of the metafor package (Harrer et al., 2022). To test for publication bias, we created funnel plots for visual inspection, with asymmetric plots indicating a skewed distribution of effect sizes (Higgins et al., 2019; Rothstein et al., 2005).

## RESULTS

The search yielded 515 results across data bases and five additional references recommended by experts, of which 244 were duplicates and thus removed for further review (see Figure 1). The titles and abstracts of the remaining 276 studies were independently screened by two reviewers with substantial agreement (κ = .71), leading to sample of 67 reports for full text screening. Thereof, 16 reports provided sufficient information for a quantitative meta‐analysis. In this step, raters achieved moderate agreement on study inclusion (κ = .59). Differences were discussed and resolved in a consensus decision. The meta‐analysis considered studies that provided quantitative data of at least pre‐ and posttest of EMIs targeting positive psychological outcomes well‐being, quality of life, and positive and negative affect. If studies did not use validated measures (k = 7), did not focus on any of these outcomes (k = 18), or if authors could not provide sufficient data for the analysis (k = 4), the records were excluded. Two records presented two different PPIs but used the same data base and control group; thus, a weighted mean effect of both intervention was used for the analysis to avoid multiple testing (Höer, 2020; Knoll, 2020). Since this meta‐analysis is not meant to discern between different PPIs (due to the anticipated low number of primary studies), this was not seen as an issue.

### Descriptive statistics

Overall, this meta‐analysis combines effect sizes for 16 positive psychological EMIs with *N* = 3397 participants (see Table 1). With two exceptions (Feldmann, 2013; Howells et al., 2014), the studies were published between 2018 and 2024. The mean age of participants, weighed by sample size per study, was 21.87 (SD = 13.02) years. Nine studies recruited student samples; two studies examined clinical samples with anxiety (LaFreniere & Newman, 2023) and colorectal cancer (Rocamora González et al., 2022). Most studies were conducted in Europe (k = 10) (Feldmann, 2013; Höer, 2020; Howells et al., 2014; Knoll, 2020; Küchler et al., 2023; Paz Castro et al., 2022; Pizarro‐Ruiz et al., 2021; Rocamora González et al., 2022; Tabernero et al., 2022; Tagalidou et al., 2019; van Roekel & Maciejewski, 2024), followed by the Americas (USA/Canada) (k = 5) (Al‐Refae et al., 2021; Daugherty et al., 2018; LaFreniere & Newman, 2023; Peterson et al., 2024; Rodgers et al., 2018), and Asia (k = 1) (Leng et al., 2023). Consequently, most participants self‐identified as White or Caucasian, although race/ethnicity was not assessed in all studies. The relative frequency of female participants was 69.1% across studies. Overall, the risk of bias was low for most studies (Cohen's kappa = 0.80), with incomplete reporting of randomization procedures and missing outcome data leading to higher risk of bias in some studies.

**TABLE 1:** Descriptive statistics of the included studies (k = 16) and potential moderators in the meta‐analysis of positive psychological ecological momentary interventions.

Authors	Year of publication	*n*	Moderators			
			Age (M)	Gender (female [%])	Region	Risk of bias
Total	‐	3397	21.87	69.1	5:10:1	
Al‐Refae et al.	2021	165	25.24	78.8	1	Low
Feldmann	2013	40	‐‐	75	2	High
Höer and Knoll	2020	84	36.54	89.23	2	Some concerns
Howells et al.	2014	121	40.7	86	2	High
Küchler et al.	2023	386	25.77	74.9	2	Low
LaFreniere and Newman	2023	85	18.66	90.59	1	Low
Leng et al.	2023	75	30.6	100	3	Low
Mirabito and Verhaeghen	2023	111	21.4	83.93	1	Low
Paz Castro et al.	2022	1,473	15.4	55.2	2	Low
Peterson et al.	2024	36	37.9	100	1	High
Pizarro‐Ruiz et al.	2021	164	22.06	82.93	2	Some concerns
Rocamora‐Gonzáles et al.	2022	82	65.1	35.4	2	High
Rodgers et al.	2018	251	18.36	74	1	Low
Tagalidou, Baier, and Laireiter	2019	182	24.91	85.16	2	Low
Van Roekel and Maciejewski	2024	142	20.5	83.8	2	Low

*Note*: Region: geographic study region coded as 1 = USA/Canada, 2 = Europe (including United Kingdom and Switzerland), 3 = Asia; −‐: value not reported/available.

### Outcomes and measurement instruments

Table 2 presents an overview of outcome measures as well as key content and delivery method of positive psychological EMIs in the included studies. Ten studies (Al‐Refae et al., 2021; Feldmann, 2013; Höer, 2020; Knoll, 2020; Küchler et al., 2023; Mirabito & Verhaeghen, 2023; Paz Castro et al., 2022; Peterson et al., 2024; Tagalidou et al., 2019; van Roekel & Maciejewski, 2024) examined effects on well‐being and happiness, using the Authentic Happiness inventory (Shepherd et al., 2015), the Subjective Happiness Scale (Lyubomirsky & Lepper, 1999), the Psychological Well‐Being Scale (Ryff & Keyes, 1995), the Mental Health Continuum‐Short Form (Keyes, 2002), and the WHO‐5. Quality of life and life satisfaction was measured in five studies (Feldmann, 2013; Höer, 2020; Knoll, 2020; Küchler et al., 2023; Rocamora González et al., 2022) with the Satisfaction With Life Scale (Diener et al., 1985) in four studies, and the WHOQOL‐BREF in one study (Rocamora González et al., 2022). Positive and negative affect was measured with the Positive and Negative Affect Schedule (PANAS) (Watson et al., 1988), and adapted forms like PANAS‐C10 (Rodgers et al., 2005), PANAS‐X Jovialty (LaFreniere & Newman, 2023) as well as the SPANE (Höer, 2020; Knoll, 2020), and the Positive Affect subscale of the BMSWB (Leng et al., 2023). Apart from two studies (LaFreniere & Newman, 2023; Leng et al., 2023), positive and negative affect was assessed concurrently in most studies. Hence, effects on positive affect were analyzed for seven studies, effects on negative affect in five studies (Höer, 2020; Howells et al., 2014; Knoll, 2020; Pizarro‐Ruiz et al., 2021; Rodgers et al., 2018).

**TABLE 2:** List of included studies, measurement instruments, and delivery and content of the positive psychological ecological momentary interventions.

Study	Instrument	Intervention delivery and content
**Well‐being**		
Al‐Refae et al. (2021)	PWBS	Smartphone: Mindfulness and self‐compassion
Feldmann (2013)	SHS	Daily diary: Three good things; gratitude letter (gratitude)
Höer (2020) and Knoll (2020)	AHI	Diary (digital and pen‐and‐paper): Scheduling positive activities: The puzzle of happiness (savoring); three good things (gratitude)
Küchler et al. (2023)	WHO‐5	Smartphone: Mindfulness
Paz Castro et al. (2022)	WHO‐5	Smartphone: Life skills (self‐management and social skills)
Peterson et al. (2024)	SHS	Smartphone: Gratitude diary; mindfulness meditation
Mirabito and Verhaeghen (2023)	PWBS	Smartphone: Mindfulness
Tagalidou et al. (2019)	AHI	Diary (digital): Coping, three fun things (humor); three good things (gratitude)
Van Roekel and Maciejewski (2024)	MHC‐SF	Smartphone: Mikro interventions from different areas (e.g. mindfulness, gratitude, savoring)
**Quality of life**		
Feldmann (2013)	SWLS	Diary (pen‐and‐paper): Three good things; gratitude letter (gratitude)
Höer (2020) and Knoll (2020)	SWLS	Diary (digital and pen‐and‐paper): Scheduling positive activities: The puzzle of happiness (savoring); three good things (gratitude)
Howells et al. (2014)	SWLS	Smartphone: Mindfulness
Pizarro‐Ruiz et al. (2021)	SWLS	Smartphone: Mindfulness
Rocamora González et al. (2022)	WHOQOL‐BREF	Smartphone: Mindfulness
**Positive affect**		
Höer (2020) and Knoll (2020)	SPANE	Diary (digital and pen‐and‐paper): Scheduling positive activities: The puzzle of happiness (savoring); three good things (gratitude)
Howells et al. (2014)	PANAS	Smartphone: Mindfulness
LaFreniere and Newman (2023)	PANAS‐X	Smartphone: Planning and perception of positive activities/moments (savoring)
Leng et al. (2023)	BMSWB	Smartphone: Mindfulness
Pizarro‐Ruiz et al. (2021)	PANAS	Smartphone: Mindfulness
Rodgers et al. (2018)	PANAS C‐10	Smartphone: Self‐compassion
**Negative affect**		
Höer (2020) and Knoll (2020)	SPANE	Diary (digital and pen‐and‐paper): Scheduling positive activities: The puzzle of happiness (savoring); three good things (gratitude)
Howells et al. (2014)	PANAS	Smartphone: Mindfulness
Pizarro‐Ruiz et al. (2021)	PANAS	Smartphone: Mindfulness
Rodgers et al. (2018)	PANAS C‐10	Smartphone: Self‐compassion

Abbreviations: AHI, Authentic Happiness Inventory; BMSWB, Body–Mind‐Spirit Well‐Being Inventory; MHC‐SF, Mental Health Continuum‐Short Form; PANAS, Positive and Negative Affect Scale; PANAS‐C10, PANAS for Children Short Form; PANAS‐X, Positive and Negative Affect Schedule Expanded Form: Joviality Scale; PWBS, Psychological Well‐Being Scale; SWLS, Satisfaction with Life Scale; SPANE, Scale of Positive and Negative Experience; SHS, Subjective Happiness Scale; WHO‐5, World Health Organization Well‐Being Index; WHOQOL‐BREF, World Health Organization Quality of Life Short Form.

### Content and delivery of positive psychological EMIs

Regarding content, nine studies focused on mindfulness or mindfulness‐based interventions (e.g. meditation and breathing) (Al‐Refae et al., 2021; Howells et al., 2014; Küchler et al., 2023; Leng et al., 2023; Mirabito & Verhaeghen, 2023; Peterson et al., 2024; Pizarro‐Ruiz et al., 2021; Rocamora González et al., 2022). The remaining studies investigated gratitude (Feldmann, 2013; Knoll, 2020; Tagalidou et al., 2019), enjoyment (LaFreniere & Newman, 2023; Peterson et al., 2024) or a combination of different approaches.

The intervention ranged from one to 8 weeks. Ten studies were web‐based or utilized a smartphone app (Al‐Refae et al., 2021; Howells et al., 2014; Mirabito & Verhaeghen, 2023; Paz Castro et al., 2022; Peterson et al., 2024; Pizarro‐Ruiz et al., 2021; Rocamora González et al., 2022; Rodgers et al., 2018; Tagalidou et al., 2019; van Roekel & Maciejewski, 2024), the remaining six studies implemented digital or analogue daily diaries (Feldmann, 2013; Höer, 2020; Knoll, 2020; Küchler et al., 2023; LaFreniere & Newman, 2023; Leng et al., 2023). All studies realized a pre‐ and posttest, and 10 studies also realized a follow‐up assessment. Appendix A3 presents a more extensive overview of interventions, outcomes, and study designs for each study.

### Meta‐analyses of effect sizes

For all four outcomes (well‐being, quality of life, and positive and negative affect) separate meta‐analyses were calculated (see Table 3). If at least three studies reported effects for pretest (T0) to posttest (T1) or posttest to follow‐up (T2), meta‐analysis was performed.

**TABLE 3:** Results of meta‐analyses of positive psychological ecological momentary interventions with random‐effects estimation and heterogeneity analysis.

	Random‐effects model			Heterogeneity			
	*k*	*n*	*g* (95% CI)	*p*	*Q*	*I* ^*2*^	*τ* ^*2*^
Posttest (T1)							
Well‐being	9	2,619	0.10 (−0.06–0.27)	0.19	16.95*	52.8%	0.03
Quality of life	5	491	−0.09 (−0.36–0.18)	0.40	4.69	14.6%	0.02
Positive affect	6	776	0.29 (−0.00–0.58)	0.05	10.96	54.4%	0.04
Negative affect	4	615	−0.05 (−0.54–0.43)	0.75	9.81*	69.4%	0.06
Follow‐up (T2)							
Well‐being	5	2267	0.21 (−0.09–0.51)	0.13	20.53***	80.5%	0.05
Quality of life	2	‐‐	‐‐	‐‐	‐‐	‐‐	‐‐
Positive affect	4	491	0.08 (−0.52–0.68)	0.72	9.37*	68%	0.09
Negative affect	2	‐‐	‐‐	‐‐	‐‐	‐‐	‐‐

*Notes*: *k:* Number of studies/reports; *n:* sample size; *g:* Hedge's g with 95% confidence interval; *p: p*‐value; *Q;* Cochrane's Q; *I* ^*2*^: Proportion of true heterogeneity between studies; *τ* ^*2*^: Overall heterogeneity in effect size estimates.

* *p* < .05,

** *p* < .01, and

*** *p* < .001.

**FIGURE 2:**
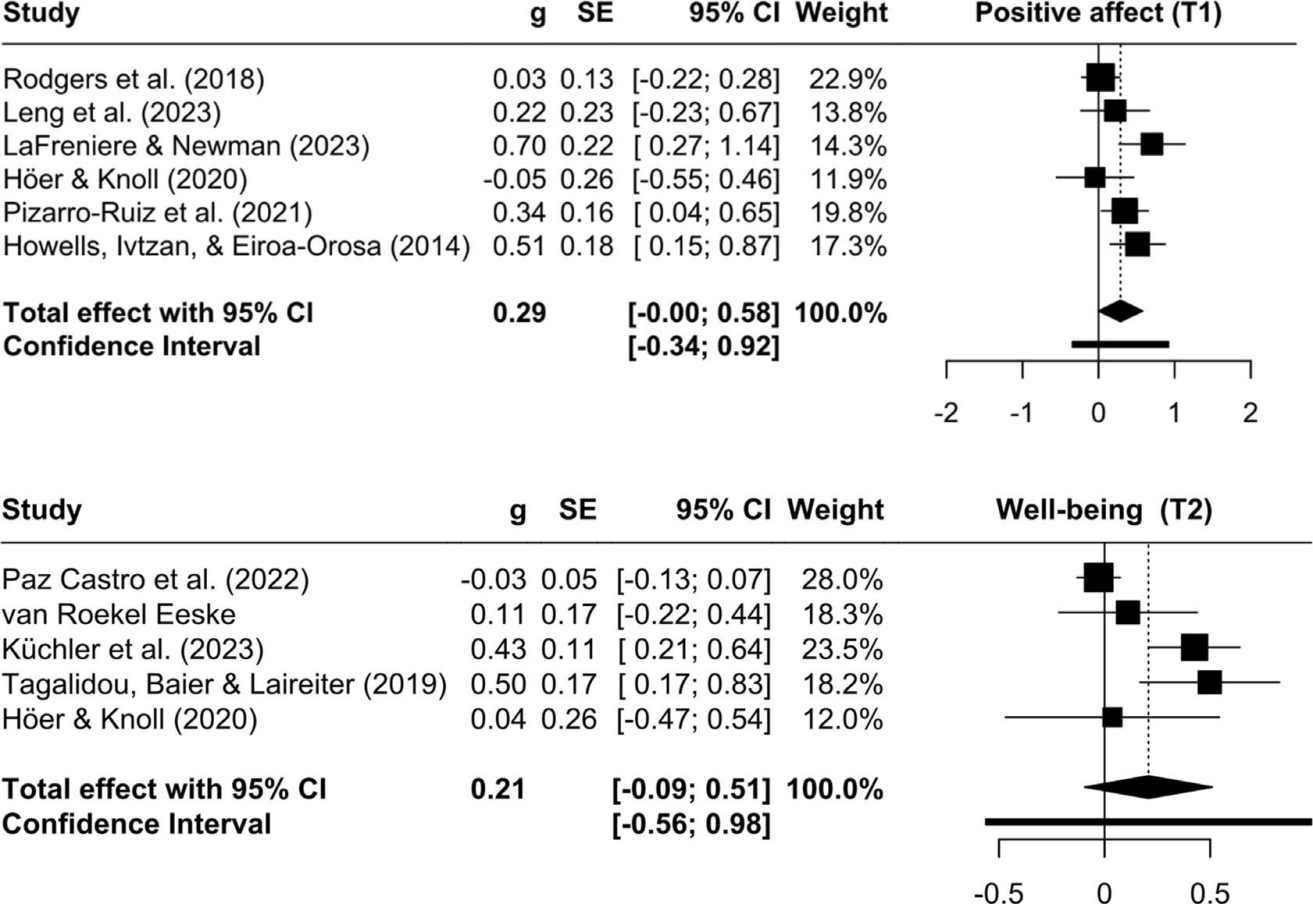
Forest plots of meta‐analysis of positive psychological ecological momentary interventions regarding positive affect (k = 6; T0 to T1) and well‐being (k = 5; T1 to T2).

Based on the random‐effect models, there was no statistically significant effect between pretest and posttest in any of the outcomes. However, a clinically significant small effect was visible for positive affect (*g* = 0.29, *p* = 0.05, *k* = 6, *n* = 776). The remaining effects were below cutoffs for small effects (Higgins et al., 2019). For quality of life, the estimated effect was negative (*g* = −0.09, *p* = 0.75, *k* = 7, *n* = 615), indicating an increase in quality of life in the control group compared to the intervention group, albeit small and with a large *p*‐value. For well‐being and positive affect, the analysis of follow‐up effects did not reach statistical significance but revealed a clinically relevant, small effect of positive psychological EMIs on well‐being (*g* = 0.21, *p* = 0.13, *k* = 4, *n* = 2267), and a smaller effect on positive affect. In comparison, the magnitude of effects was reversed, with a larger short‐term effect of EMIs on positive affect but a larger midterm effect on well‐being. Forest plots were created to illustrate the differences between studies and their impact on the effect estimates for all outcomes (see Appendix A4). Here, we present forest plots for the meta‐analysis of pre‐ and posttest for positive affect as well as posttest and follow‐up for well‐being, since these two were clinically but not statistically significant (see Figure 2).

### Heterogeneity

It can be noted that the variance and thus heterogeneity of effects is much greater for well‐being at follow‐up than for positive affect at posttest. Regardless of the type and intensity of PPIs, there seems to be a slight mood‐boosting short‐term effect, but effects differ for follow‐up effects and well‐being as an outcome. Heterogeneity between studies was substantial for well‐being (Q = 16.95, *p* = 0.03) and negative affect (Q = 9.81, *p* = 0.02) at T1 as well as well‐being (Q = 20.53, *p* < .001) and positive affect (Q = 9.37, *p* = 0.02) at T2. This was supported by *I* ^2^ values that indicated moderate to high heterogeneity except for quality of life at T1 (*I* ^2^ = 14.6%). Heterogeneity seemed to increase over time, as both values at T2 were descriptively higher than at T1.

### Sensitivity analysis and potential publication bias

To examine potential outliers, the metafor package in R (Harrer et al., 2022) was used. Via Baujat plots (see Appendix A5) and subsequent sensitivity analyses with and without potential outliers, meta‐analytical models were re‐examined. Neither of the models achieved statistical significance, but the random‐effect models were closer to statistical significance for well‐being at T1 (*g* = 0.06, *p* = 0.08) and at T2 (*g* = 0.32, *p* = 0.06) when excluding potential outliers. Although the number of primary studies was very low, the results show a similar pattern, namely, a small short‐term effect of positive psychological EMIs on well‐being, but by trend, a larger effect at follow‐up.

The risk of publication bias was approximated via visual inspection of funnel plots (see Appendix A6) due to a low sample size (*k* < 10) prohibiting more elaborate estimations (Borenstein et al., 2021; Egger et al., 1997). For T1, funnel plots seem symmetrical for quality of life, positive, and negative affect but slightly skewed toward the control group for well‐being. For T2, funnel plots seem symmetrical for well‐being and positive affect.

### Moderator analysis

To inspect potential moderators of effect sizes, age, gender, study region, and risk of bias were examined in subgroup analyses and meta‐regression models for estimations with statistically significant Q values and thus heterogeneity (see Table 3 and Appendix A7). Accordingly, these models were calculated for well‐being and negative affect at T1 as well as well‐being and positive affect at T2. It is recommended to test for moderator effect if at least 10 primary studies are available per outcome, so the analysis is rather exploratory (Higgins & Thompson, 2002). None of the moderators reached statistical significance, but the largest impact was observed for gender and positive affect at T2, with potentially stronger effects of EMIs for women compared to men. In addition, studies with lower risk of bias by trend showed stronger effects of EMIs on positive affect.

## DISCUSSION

This meta‐analysis synthesizes the empirical evidence on PPIs delivered as EMIs. It complements the rich literature on PPIs and EMIs by taking a closer look at the combination of the two. The analysis focused on four key outcomes of PPIs, namely, well‐being, quality of life, and positive and negative affect and explored the impact of positive psychological EMIs in short‐term (pre/post) and midterm perspectives (follow‐up). Moreover, study region, age, gender, and risk of bias were considered as moderators of meta‐analytic effects.

Overall, we did not find any statistically significant effects of EMIs, but clinically relevant differences in favor of PPIs compared to controls for positive affect (posttest) and well‐being (follow‐up). The differences in negative affect and quality of life were even smaller and seemed to be inconsistent. Neither of the explored moderators was significant, but women seemed to benefit more strongly than men regarding positive affect (at posttest), and studies of higher quality seemed to achieve stronger effects.

While this does not correspond to many previous analyses of traditional PPIs (i. e. PPIs without a focus on EMIs) (Bolier et al., 2013; Carr et al., 2023; Proyer et al., 2015), it also points to differences in short‐term to mid‐/long‐term impact of PPIs. Frameworks like broaden‐and‐build (Fredrickson, 2004) assume that repeated positive psychological practice helps to shift to a more positive perspective that more frequently considers positive cognitions, emotions, and behaviors when confronted with challenging situations and decision‐making tasks. In this sense, it takes time to create positive habits and achieve an impact on well‐being, whereas creating positive affect is easier to achieve but also may fluctuate more across time (i.e. short‐term effects but no long‐term stability). This assumption reflects our findings and also corresponds to previous observations, for instance, regarding the *best possible self*‐writing exercise, where participants are asked to write about their best future selves (Carrillo et al., 2019; Heekerens & Eid, 2021; Tomczyk et al., 2023). Across many studies, the writing exercise had small short‐term effects on positive affect and with continued practice, showed more positive mid/long‐term effects on other outcomes like well‐being. This is promising for positive psychological practice in nonclinical populations, since it indicates that mobile‐based daily practice might be useful to increase positive mood and boost resilience in the long‐term regardless of the extent of current stressors (e.g. mental illness; as suggested by Waters et al., 2022). This has a broad appeal for public mental health, since mobile‐based interventions have a low barrier to entry for most people and can easily be integrated into preexisting routines. Nevertheless, we found few studies of mixed quality. More EMA and EMI research is needed to examine day‐to‐day changes and fluctuations in positive psychological outcomes. Similarly, in research on self‐compassion or psychotherapy, mostly with clinical samples, confronting negative emotions or experiences and shifting toward more positive perspectives can lead to an initial decline in well‐being or quality of life, but it has a beneficial long‐term effect if persons can build functional cognitions, for instance, by reframing their experience, and experience successful coping with these stressors (Ewert et al., 2021; Linden et al., 2018). However, due to the small number of primary studies and the heterogeneity in PPI design and delivery, it was not possible to take a closer look at type and duration of PPI and its impact on short‐ and midterm changes in outcomes. Yet, our estimation yielded more robust findings for outcomes with multiple primary studies. Consequently, more primary studies and a more standardized approach to evaluation design and assessment schedules are recommended.

Moreover, previous systematic reviews and meta‐analyses of PPIs indicate that participants who are more strongly affected by mental health burden (e.g. clinical samples) may also benefit more from PPIs (Carr et al., 2021, 2023; Sin & Lyubomirsky, 2009). Since we did not limit our analysis to clinical samples and only two studies implemented positive psychological EMIs in clinical samples (LaFreniere & Newman, 2023; Rocamora González et al., 2022), this is a question for future research. Therefore, more studies should test the impact of PPIs and positive psychological EMIs, across a greater variety of mental health burden (e.g. population samples with elevated levels of depression or anxiety, patients with clinically diagnosed mental disorders). We also did not find many studies with children or adolescents that could have been included in the analysis, underlining the need for more research and development in these groups.

Similarly, the exploration of potential moderators did not yield statistically significant results, and it seemed that studies with lower risk of bias reported stronger effects on positive affect. The recommended number of 10 or more studies for moderator analysis (Borenstein et al., 2021) was not reached, and the studies were rather homogeneous regarding age, gender, and sociodemographic background (mostly White, younger, female university/college students). There also was a trend for stronger effects on positive affect in women compared to men. While this observation is not uncommon in psychological research, it is problematic for external validity, and interpreting global effects of PPIs and positive psychological EMIs. The positive psychology research community is aware of these issues (van Zyl et al., 2024; van Zyl & Rothmann, 2019; Wissing, 2022), yet there is still a long way to go to reach more diverse samples, explore efficacy and effectiveness of interventions in different contexts and with different outcomes and utilizing psychometrically validated measures. The analysis of a potential publication bias and the impact of outliers showed that there were no strong indications for a publication bias in favor of PPIs, but several studies had strong individual impacts on effect size estimations. With a larger set of primary studies, this effect might be diminished, leading to a more balanced result. Since this is an emerging field (most studies were published between 2018 and 2024), we assume that the evidence base will grow considerably within the next few years.

### Strengths and limitations

This meta‐analysis has several challenges: First, there were a low number of primary studies (a number of 5–10 studies per effect size is recommended) (Borenstein et al., 2021). In accordance with Ewert et al. (2021), we included outcomes with at least three primary studies, with most studies reporting effects for well‐being and positive affect at posttest (T1). Hence, these estimated effects are more precise and reliable than the others. Second, although funnel plots did not indicate a strong publication bias, it is important to consider gray literature and potentially (peer‐reviewed) preprints to reduce publication bias. We have contacted experts via scientific associations and also included reports without a traditional peer review (e.g. Master's thesis) to address this issue. However, this might have been a trade‐off with risk of bias or study quality in some cases. Our findings include studies with mixed risk of bias, which might limit the generalizability of the results. Indeed, gray literature received higher risk of bias scores than most peer‐reviewed publications. In recent years, the Open Science movement has advocated for Good Scientific Practice and Open Science practices in education (Klein et al., 2018), which might also be promising for positive psychology scholarship (van Zyl et al., 2024). Third, including studies with varying methods (e.g. different outcomes and outcome measures) and designs (e.g. EMI format and delivery method) figuratively compares apples and oranges in the analysis. While we aimed for a minimum standard of positive psychological EMI and control group, quantitative pre‐ and posttest in each primary study, and grouped similar constructs and measures, there were still differences in the type of population, the intervention and the assessment schedule. Moreover, some constructs like positive affect and life satisfaction (which can be considered a part of quality of life) are statistically correlated, challenging their conceptual clarity (Medvedev & Landhuis, 2018; Shepherd et al., 2015). We observed moderate to high heterogeneity for most estimations, similar to previous work (Bolier et al., 2013). For effect size estimation, we utilized change scores and Hedge's g to account for small samples and baseline differences between groups, which was comparable to previous research (Bolier et al., 2013; Carr et al., 2023; Carrillo et al., 2019; Heekerens & Eid, 2021; Proyer et al., 2015).

### Implications for future research

The analysis shows that there is potential, yet not enough evidence, of the efficacy of positive psychological EMIs regarding well‐being in the general population. In particular, clinically significant changes in positive affect and well‐being hint at their potential. Future research should aim for more diverse samples, including Non‐Western, LGBTQIA+, and other marginalized communities (Duan et al., 2022), focus on different aspects of EMIs and their impact (e.g. frequency, delivery method, duration, and elements of gamification), and compare specific (i.e. focused on specific topics or target groups) and more generic EMIs (i.e. combining different PPIs). For instance, the literature on (mobile‐based) mindfulness interventions indicates a positive effect of mindfulness on stress, negative emotions, and well‐being (Allen et al., 2021; Tabernero et al., 2022; Zheng et al., 2022). However, the low number of EMIs studies do not allow for an in‐depth comparisons of different PPIs and their effects. Moreover, blended care (or blended prevention) approaches were hardly present in the included studies. While integrating face‐to‐face and online interventions is already an important part of mental health care and mobile health interventions across the globe (Owusu et al., 2022; Wentzel et al., 2016), this is not necessarily the case for positive psychology or (positive) public mental health outside of clinical contexts. Therefore, conducting effectiveness studies and real‐world implementation of such EMIs is an important next step in examining their impact.

## CONCLUSION

PPIs hold promise for boosting well‐being, quality of life and positive affect in diverse populations, and thus positive public mental health. However, despite their popularity, not much is known about the efficacy of PPIs delivered as EMIs (e.g. via mobile applications) in daily life. Therefore, this meta‐analysis synthesizes the literature on positive psychological EMIs via random‐effects models, comparing interventions with control groups (active or passive). While we did not find any statistically significant effect for the four outcomes quality of life, well‐being, and positive and negative affect, nor a moderator effect of study region, age, gender, or risk of bias, there were clinically significant changes in favor of the intervention for positive affect at posttest and well‐being at follow‐up. The results support the notion of mood‐boosting short‐term effects of PPIs and potential long‐term benefits for well‐being. The low number of primary studies, the mixed risk of bias across primary studies, and substantial heterogeneity of effects made it difficult to achieve robust and reliable estimates. Therefore, to fully realize their potential and generate evidence for positive psychological EMIs, future research should aim to utilize standardized outcome measures, adhere more strongly to reporting guidelines, and examine more diverse and larger samples to improve internal and external validity.

## CONFLICT OF INTEREST STATEMENT

None.

## ETHICS STATEMENT

This study is a secondary data analysis (meta‐analysis) of previous work, therefore it is exempt from ethical review.

## Supporting information


**Appendix A1:** Search terms and search strings
**Appendix A2:** List of included studies, key outcomes and measurement instruments
**Appendix A3:** Description of positive psychological ecological momentary interventions
**Appendix A4:** Forest plots of meta‐analytic estimates for all outcomes (well‐being, quality of life, positive affect, and negative affect) at posttest (T1) and follow‐up (T2)
**Appendix A5:** Baujan plots of meta‐analytic estimates to identify influential outliers
**Appendix A6:** Funnel plots of meta‐analytic estimates for all outcomes (well‐being, quality of life, positive affect, and negative affect) at posttest (T1) and follow‐up (T2)
**Appendix A7:** Results of subgroup analysis and meta‐regression models with moderators age, gender, study region and risk of bias score

## Data Availability

The study was preregistered at PROSPERO: CRD42024528187; https://www.crd.york.ac.uk/prospero/display_record.php?ID=CRD42024528187. Supporting informations are available at the Open Science Framework: https://osf.io/8qhwk/?view_only=ea175ea092c44b499249be8b221a34bd.
