# Supplementary material for: Positive changes in daily life? A meta‐analysis of positive psychological ecological momentary interventions
Source: Appl Psychol Health Well Being. 2025 Feb 10;17(1):e70006. doi: 10.1111/aphw.70006 (PMC11811679; doi:10.1111/aphw.70006)
Supplement: Supplementary file 1 — Appendix A1: Search terms and search strings Appendix A2: List of included studies, key outcomes and measurement instruments Appendix A3: Description of positive psychological ecological momentary interventions Appendix A4: Forest plots of meta‐analytic estimates for all outcomes (well‐being, quality of life, positive affect, and negative affect) at posttest (T1) and follow‐up (T2) Appendix A5: Baujan plots of meta‐analytic estimates to identify influential outliers Appendix A6: Funnel plots of meta‐analytic estimates for all outcomes (well‐being, quality of life, positive affect, and negative affect) at posttest (T1) and follow‐up (T2) Appendix A7: Results of subgroup analysis and meta‐regression models with moderators age, gender, study region and risk of bias score [file APHW-17-0-s001.docx]

**Appendix**

Appendix A.1: Search terms and search strings

Appendix A.2: List of included studies, key outcomes and measurement instruments

Appendix A.3: Description of positive psychological ecological momentary interventions

Appendix A.4: Forest plots of meta-analytic estimates for all outcomes (well-being, quality of life, positive affect, and negative affect) at posttest (T1) and follow-up (T2)

Appendix A.5: Baujan plots of meta-analytic estimates to identify influential outliers

Appendix A.6: Funnel plots of meta-analytic estimates for all outcomes (well-being, quality of life, positive affect, and negative affect) at posttest (T1) and follow-up (T2)

Appendix A.7: Results of subgroup analysis and meta-regression models with moderators age, gender, study region and risk of bias score

# Appendix A.1

**Table A.1.1**

*Search terms used for identifying positive psychological ecological momentary interventions efficacy and effectiveness reports*

|  | (1) EMI | (2) PPI |
| --- | --- | --- |
| Search terms | EMI  EMA  mHealth  mobile technolog*  palmtop computer*  mobile phone*  sensor*  experience sampl*  event-contingent sampl*  written diar*  electronic diar*  online diar*  daily diar* |  |
|  |  |  |
| Combination of | momentary  mobile*  ambulatory  smartphone*  telephone*  *und*  intervention  assessment | positive  positive psycholog*  ppi  savoring  gratitude  kindness  empathy  optimism  strenght*  meaning  positive emotion*  engagement  positive relationship*  accomplishment  forgiveness  goal*  value*  best possible self  imagined self technique  positive writing  positive thinking  positive reframing  positive affirmation  journaling  hope  three good things  counting blessings  self-reflection  mindfulness  self-compassion  self-acceptance  *und*  intervention  treatment  therapy  training  exercise  prevention |

Notes. ^*^ Truncation, EMI Ecological Momentary Intervention, PPI Positive Psychological Intervention

**Table A.1.2**

*Search strings and limitations of each data base search*

| APA PsycInfo | |
| --- | --- |
| Limitations | Search terms in title or abstract  Year of publication: 2005 - 2024  Language: German or English |
| Search | ((TI EMI OR TI EMA OR TI mHealth OR TI mobile technolog* OR TI palmtop computer* OR TI mobile phone* OR TI sensor* OR TI experience sampl* OR TI event-contingent sampl* OR TI written diar* OR TI electronic diar* OR TI online diar* OR TI daily diar*) OR ((TI momentary OR TI mobile* OR TI ambulatory OR TI smartphone* OR TI telephone*) AND (TI intervention OR TI assessment))) AND ((TI positive OR TI positive psycholog* OR TI ppi OR TI savoring OR TI gratitude OR TI kindness OR TI empathy OR TI optimism OR TI strenght* OR TI meaning OR TI positive emotion* OR TI engagement OR TI positive relationship* OR TI accomplishment OR TI forgiveness OR TI goal* OR TI value* OR TI best possible self OR TI imagined self technique OR TI positive writing OR TI positive thinking OR TI positive reframing OR TI positive affirmation* OR TI journaling OR TI hope OR TI three good things OR TI counting blessings OR TI self-reflection OR TI mindfulness OR TI self-compassion OR TI self-acceptance) AND (TI intervention OR TI treatment OR TI therapy OR TI training OR TI exercise OR TI prevention)) |
| Pubmed | |
| Limitations | Search terms in title or abstract  Year of publication: 2005 - 2024  Language: German or English |
| Search | ((EMI[Title] OR EMA[Title] OR mHealth[Title] OR mobile technolog*[Title] OR palmtop computer*[Title] OR mobile phone*[Title] OR sensor*[Title] OR experience sampl*[Title] OR event-contingent sampl*[Title] OR written diar*[Title] OR electronic diar*[Title] OR online diar*[Title] OR daily diar*[Title]) OR ((momentary[Title] OR mobile*[Title] OR ambulatory[Title] OR smartphone*[Title] OR telephone*[Title]) AND (intervention[Title] OR assessment[Title]))) AND ((positive[Title] OR positive psycholog*[Title] OR ppi[Title] OR savoring[Title] OR gratitude[Title] OR kindness[Title] OR empathy[Title] OR optimism[Title] OR strenght*[Title] OR meaning[Title] OR positive emotion*[Title] OR engagement[Title] OR positive relationship*[Title] OR accomplishment[Title] OR forgiveness[Title] OR goal*[Title] OR value*[Title] OR best possible self[Title] OR imagined self technique[Title] OR positive writing[Title] OR positive thinking[Title] OR positive reframing[Title] OR positive affirmation*[Title] OR journaling[Title] OR hope[Title] OR three good things[Title] OR counting blessings[Title] OR self-reflection[Title] OR mindfulness[Title] OR self-compassion[Title] OR self-acceptance[Title]) AND (intervention[Title] OR treatment[Title] OR therapy[Title] OR training[Title] OR exercise[Title] OR prevention[Title])) |
| Web Of Science | |
| Limitations | Search terms in title or abstract  Year of publication: 2005 - 2024  Language: German or English |
| Search | TI=(((EMI OR EMA OR mHealth OR mobile technolog* OR palmtop computer* OR mobile phone* OR sensor* OR experience sampl* OR event-contingent sampl* OR written diar* OR electronic diar* OR online diar* OR daily diar*) OR ((momentary OR mobile* OR ambulatory OR smartphone* OR telephone*) AND (intervention OR assessment))) AND ((positive OR positive psycholog* OR ppi OR savoring OR gratitude OR kindness OR empathy OR optimism OR strenght* OR meaning OR positive emotion* OR engagement OR positive relationship* OR accomplishment OR forgiveness OR goal* OR value* OR best possible self OR imagined self technique OR positive writing OR positive thinking OR positive reframing OR positive affirmation* OR journaling OR hope OR three good things OR counting blessings OR self-reflection OR mindfulness OR self-compassion OR self-acceptance) AND (intervention OR treatment OR therapy OR training OR exercise OR prevention))) |

# Appendix A.2

**Table A.2**

*List of included studies, measurement instruments and delivery and key targets of the positive psychological ecological momentary interventions*

| Study | Instrument | Intervention delivery and key targets |  |
| --- | --- | --- | --- |
| **Well-being** | | | |
| Al-Refae et al. (2021) | PWBS | Smartphone: Mindfulness and self-compassion |  |
| Feldmann (2013) | SHS | Daily Diary: Three good things; gratitude letter (gratitude) |  |
| Höer (2020) & Knoll (2020) | AHI | Diary (digital and pen-and-paper): Scheduling positive activities: the puzzle of happiness (savoring); Three good things (gratitude) |  |
| Küchler et al. (2023) | WHO-5 | Smartphone: Mindfulness |  |
| Paz Castro et al. (2022) | WHO-5 | Smartphone: Life skills (self-management & social skills) |  |
| Peterson et al. (2024) | SHS | Smartphone: Gratitude diary; mindfulness meditation |  |
| Mirabito & Verhaeghen (2023) | PWBS | Smartphone: Mindfulness |  |
| Tagalidou, Baier & Laireiter (2019) | AHI | Diary (digital): Coping, Three fun things (humor); three good things (Gratitude) |  |
| Van Roekel & Maciejewski (in press) | MHC-SF | Smartphone: Mikro interventions from different areas (e.g., mindfulness, gratitude, savoring) |  |
| **Quality of life** | | | |
| Feldmann (2013) | SWLS | Diary (pen-and-paper): Three good things; gratitude letter (gratitude) |  |
| Höer (2020) & Knoll (2020) | SWLS | Diary (digital and pen-and-paper): Scheduling positive activities: the puzzle of happiness (savoring); Three good things (gratitude) |  |
| Howells et al. (2014) | SWLS | Smartphone: Mindfulness |  |
| Pizarro-Ruiz et al. (2021) | SWLS | Smartphone: Mindfulness |  |
| Rocamora González et al. (2022) | WHOQOL-BREF | Smartphone: Mindfulness |  |
| **Positive affect** | | | |
| Höer (2020) & Knoll (2020) | SPANE | Diary (digital and pen-and-paper): Scheduling positive activities: the puzzle of happiness (savoring); Three good things (gratitude) |  |
| Howells et al. (2014) | PANAS | Smartphone: Mindfulness |  |
| LaFreniere & Newman (2023) | PANAS-X | Smartphone: Planning and perception of positive activities/moments (savoring) |  |
| Leng et al. (2023) | BMSWB | Smartphone: Mindfulness |  |
| Pizarro-Ruiz et al. (2021) | PANAS | Smartphone: Mindfulness |  |
| Rodgers et al. (2018) | PANAS C-10 | Smartphone: Self-compassion |  |
| **Negative affect** | | | |
| Höer (2020) & Knoll (2020) | SPANE | Diary (digital and pen-and-paper): Scheduling positive activities: the puzzle of happiness (savoring); Three good things (gratitude) |  |
| Howells et al. (2014) | PANAS | Smartphone: Mindfulness |  |
| Pizarro-Ruiz et al. (2021) | PANAS | Smartphone: Mindfulness |  |
| Rodgers et al. (2018) | PANAS C-10 | Smartphone: Self-compassion |  |

Note. Psychological Well-Being Scale (PWBS), Subjective Happiness Scale (SHS), Authentic Happiness Inventory (AHI), Mental Health Continuum Short Form (MHC-SF), World Health Organization Well-Being Index (WHO-5), Satisfaction with Life Scale (SWLS), World Health Organization Quality of Life Short Form (WHOQOL-BREF), Scale of Positive and Negative Experience (SPANE), Positive and Negative Affect Scale (PANAS), Positive and Negative Affect Schedule Expanded Form: Joviality Scale (PANAS-X), Body-Mind-Spirit Well-Being Inventory (BMSWB), PANAS for Children Short Form (PANAS-C10)

# Appendix A.3

**Table A.3**

*Description of positive psychological ecological momentary interventions in the included studies (k = 16)*

| **Study** | **Name of the application/**  **intervention** | **Type of intervention** | **Target group** | **Material**  **/Device** | **Process/Content** | | | **Modality/**  **Format** | | **Location** | | **Duration** | | **Adherence** | |
| --- | --- | --- | --- | --- | --- | --- | --- | --- | --- | --- | --- | --- | --- | --- | --- |
| Al-Rafaen et al. (2021) | Serene | mindfulness- and self-compassion-based cognitive intervention | > 18 years | app | psychoeducation on the benefits of mindfulness and self-compassion; dealing with unexpected distress or upsetting feelings; cognitive restructuring, its benefits, and examples for each step in this process; benefits and examples of healthy coping strategies; techniques for mindful journaling and self-compassionate writing; mindfulness meditations, nature sounds and music playlists | | | smartphone app | | online | | daily, 4-week | | engagement with the app | |
| Feldmann (2013) | three good things & gratitude letter | positive psychological interventions | university students | powerpoint presentation, copied instructions for the letter of gratitude | three good things, writing and presenting the letter of gratitude | | | presentation, pen and paper | | first meeting in-person at a university course; daily in the evening at home (three good things); writing and presenting one gratitude letter | | daily, 2-week | | frequency of the completion of the task three good things; presentation of the gratitude letter | |
| Höer (2020) | Patiently Puzzled Happiness | positive psychological interventions | NR | exercise book (early memories, patiently puzzled happiness, three good things) | decide in the morning for one feel-good activty for the day | | | daily activities | | online | | daily, 1-week | | self-report of frequency and how exactly the instruction was followed | |
| Howells et al. (2014) | the "app" in happiness / Headspace On-The-Go | mindfulness-based intervention | > 18 years | app | simple daily activities based on mindfulness practice; basic concepts of mindfulness through simple guided meditations | | | smartphone app | | online | | daily, 10 min/10 days | | NR | |
| Knoll (2020) | PPI-Tree-Good-Things | positive psychological interventions | > 18 years | exercise book (early memories, patiently puzzled happiness, three good things) | three good things | | | pen and paper | | online | | daily, 1-week | | NR | |
| Küchler et al. (2023) | StudiCare Mindfulness | internet- and mobil-based interventions | college students, > 18 years, German langage speakers, internet access, moderate to low mindfulness | app | guided (on demand) or unguides intervention; provided information on stress, well-being, and mindfulness with a different focus each week (e.g., interoception, dysfunctional thinking, values and goals); weekly alternating, mindfulness exercises such as body scans and breathing meditations; homework (audio, mindfulness diary); general stress managment techniques | | | smartphone app | | online | | 8-week to 6-month, 45-60 min/week | | NR | |
| LaFreniere & Newman (2023) | SkillJoy | ecological momentary interventions | 18 to 24 years, person with GAD | app | prompts; attend to positive aspects of the present moment, engage in and savor planned enjoyable activities, record and reflect on recent positive experiences, note events that turned out well, and look forward to positive events | | | smartphone app | | online | | daily, 1-week | | NR | |
| Leng et al. (2023) | Thriving in Pregnancy - Cultivating the Four Immeasurables | mindfulness-based interventio | perinatal women experience heightened stress in a community sample | app, virtual group | alleviating negative emotional experiences, ating the four positive attitudes towards oneself and others; App module self-learning of the didactic teaching through a mini-program we developed embedded in a Chinese social media App-WeChat, and virtual group sharing of meditation practice experiences and reflection on contemplative questions | | | smartphone app, discussion | | online | | daily (app + 20-40 min mditation), 8-week (1 time a week online meeting) | | participation in the sharing of meditation experiences and reflective questions on the virtual WeChat group | |
| Mirabito & Verhaeghen (2023) | Koru Mindfulness Intervention | mindfulness-based intervention | college students | app | complete 10 minutes of formal practice per day using one of these techniques (includes body scan meditation, breathing meditation, belly breathing, dynamic breathing, gatha meditation, labeling-of-thought and labeling-of-feeling meditation, and walking meditation); every week, they also picked an everyday activity (such as brushing their teeth, taking a shower, or drinking their morning coffee) to perform mindfully; wrote a daily brief reflection; online meeting | | | smartphone app | | online | | daily (10 min + weekly everyday activity to perform indfully + brief reflecion), 4-week | | quality of mindfulness practice, quality of informal practice, exercise quantity | |
| PazCastro et al. (2021) | SmartCoach | life skills intervention | adolescents, students in secondary and upper secondary schools, > 14 years | app, website | tailored web-based feedback to reduce the individual level of stress, which was delivered directly after completion of the baseline assessment, and tailored mobile phone SMS text messages to promote self-management skills (block 1), social skills (block 2), and substance use resistance skills (block 3); one SMS text message prompt per week; contests | | | smartphone, SMS, website | | online | | 6 month | | total number of interactions; the number of responses to the weekly SMS text message prompts (quizzes, self-challenges, and individual stress and skills trainings); the number of retrieved media objects within the program (videos, pictures, and website links); and the number of views, posts, and votes within contests | |
| Peterson et al. (2024) | GEM app | positive psychological interventions | > 18 years, nursing/healthcare staff, all women | app | The GEM app provided daily notification reminders, the participants could use the app for up to 21 consecutive days | | | smartphone app | | online | | 21 days | | Dropout, incomplete outcome data, days of app use | |
| Pizarro-Ruiz et al. (2021) | Aire Fresco (Fresh Air) app (guided mindfulness) | mindfulness-based intervention | > 18 years, university students | app | The app provides guided mindfulness sessions and participants were informed via e-mail to do one session per day for the next 14 days | | | smartphone app | | oline | | 14 days | | NR | |
| Rocamora González et al. (2022) | Calm in the Operating Room app | mindfulness-based intervention | Patients with colorectal cancer waiting for surgery, having a severe mental disorder diagnosis | app | mindfulness exercises for patients with a short form (a few hours or days before surgery) and a long form (15 days to a month before surgery) | | | smartphone app | | online | | self-administered, between 15 days before surgery (T0) and one month after discharge (T2) | | Dropout, incomplete outcome data, days of app use | |
| Rodgers et al. (2018) | BodiMojo | | > 14 years, late adolescents, emerging adults | app | twice daily text messages, mood tracking and emotion regulation, gratitude journaling | | | smartphone app | | online | | 6 weeks | | NR | |
| Tagalidou et al. (2019) | Three funny things, coping humor | humor-based interventions (three funny things, coping humor, three good things), early memories | > 18 years, no current psychotherapeutic treatment and no use of psychotropic drugs or illegal drugs in the last six months; university students | website | website | online | 7 days | | NR | | | | NR | | |
| Van Roekel & Maciejewski (in press) | Microinter | microinterventions for low and high positive emotions (<20 and >80 percentile cut-off) | psychology students | app | based on a 15-day assessment period, followed by a 6-day break, a within person positive emotion score was calculated, followed by a 15-day intervention period with up to two interventions per day if the cut-off was met (low: mindfulness, acts of kindness, behaviro activation, positive mental time travel; high: savoring, social sharing, gratitudes, acts of kindness) | | | NR | | | NR | | | |  |

*Note.* GAD: generalized anxiety disorder; ICD: International Classification of Diseases; NR: not reported; PPI: positive psychological intervention

# Appendix A.4

Forest plots of meta-analytic estimates for all outcomes (well-being, quality of life, positive affect, and negative affect) at posttest (T1) and follow-up (T2)

**Figure A.4.1**

*Forest plot of positive psychological EMI effects on well-being at posttest (T1)*





**Figure A.4.2**

*Forest plot of positive psychological EMI effects on quality of life at posttest (T1)*





**Figure A.4.3**

*Forest plot of positive psychological EMI effects on positive affect at posttest (T1)*


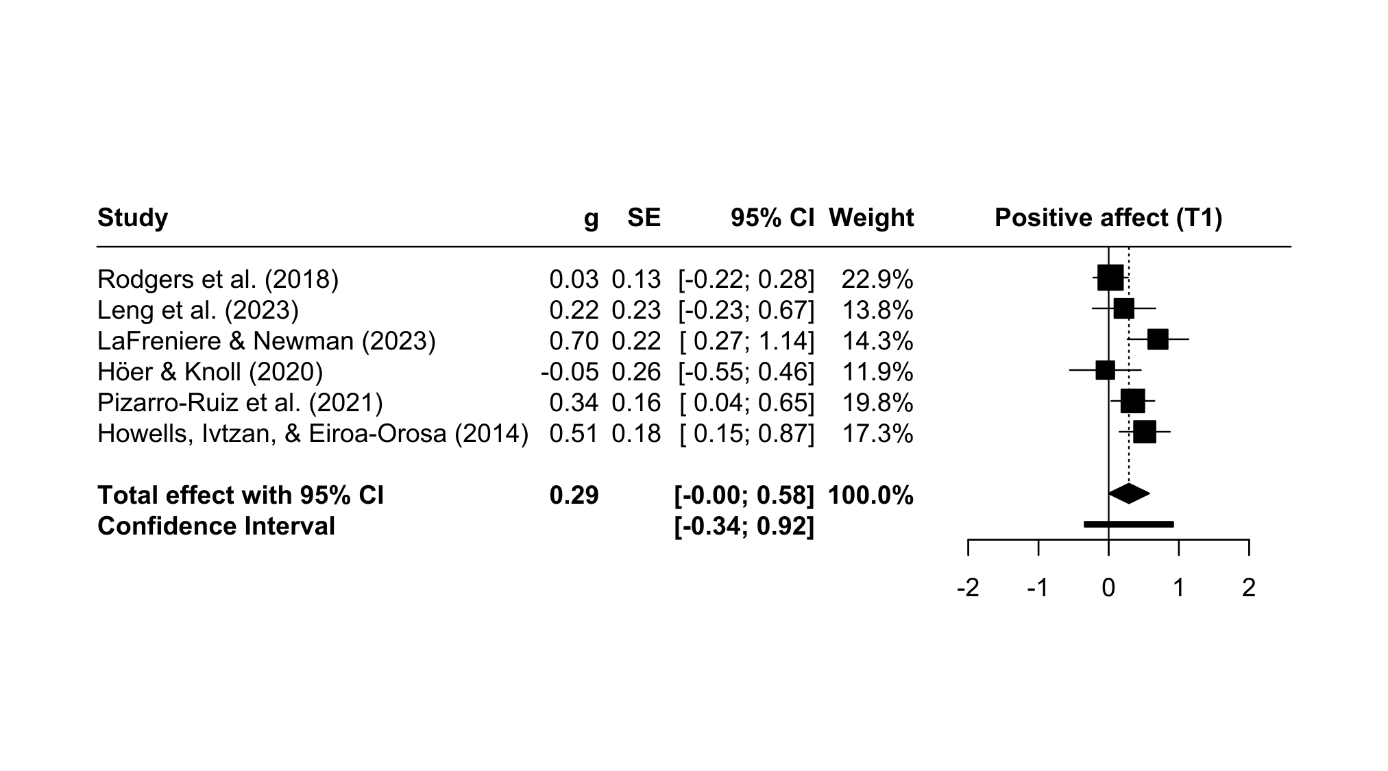


**Figure A.4.4**

*Forest plot of positive psychological EMI effects on negative affect at posttest (T1)*


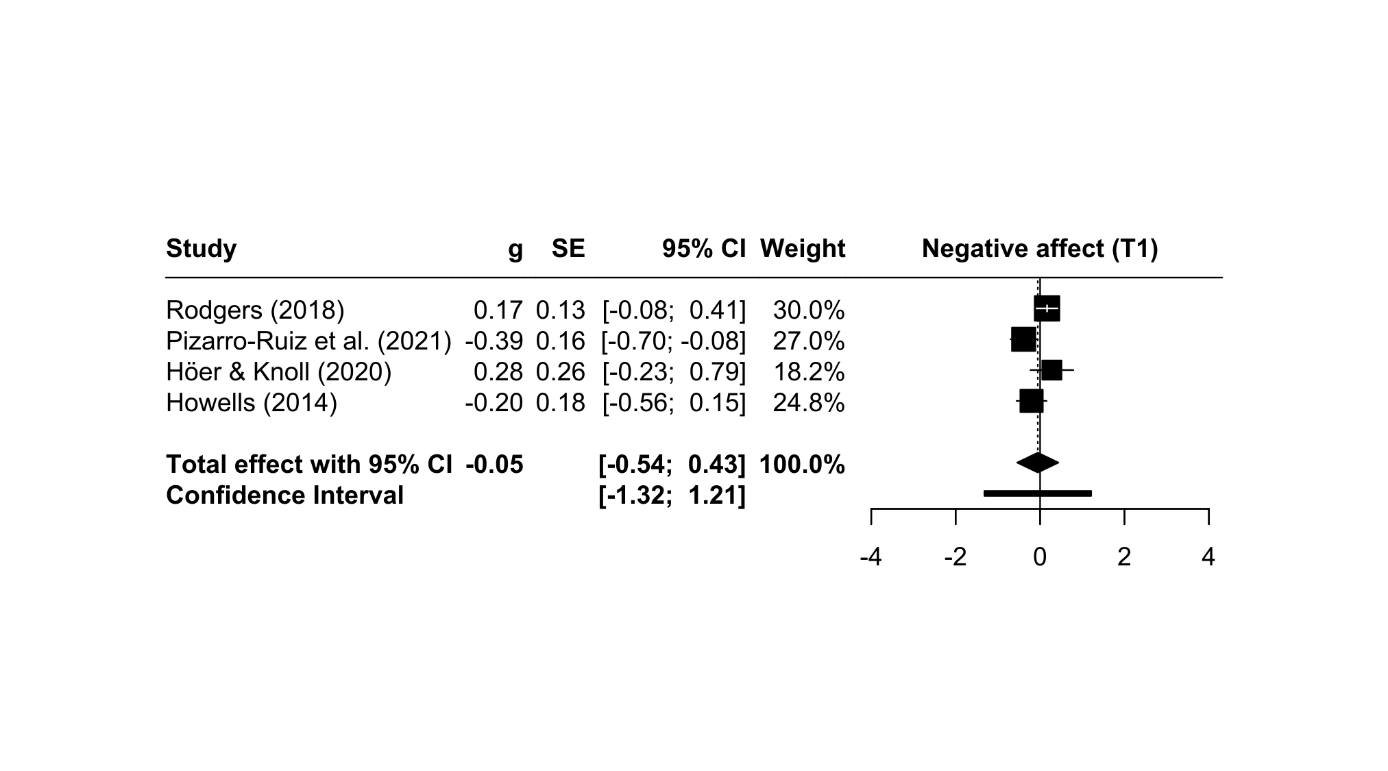


**Figure A.4.5**

*Forest plot of positive psychological EMI effects on well-being at follow-up (T2)*

*

*

**Figure A.4.6**

*Forest plot of positive psychological EMI effects on positive affect at follow-up (T2)*

*

*

# Appendix A.5

Baujat plots to identify influentia outliers with strong impact on the total effect size

**Figure A.5.1**





*Baujat plots of outcomes with significant heterogeneity at post-test (T1), namely well-being (left plot) and negative affect (right plot)*

*

***Figure A.5.2**

*

Baujat plots of outcomes with significant heterogeneity at follow-up (T2), namely well-being (left plot) and positive affect (right plot)*

# Appendix A.6

Funnel plots of meta-analytic estimates for all outcomes (well-being, quality of life, positive affect, and negative affect) at posttest (T1) and follow-up (T2)

**Figure A.6.1**

*Funnel plot of included studies on well-being at posttest (T1)*





**Figure A.6.2**

*Funnel plot of included studies on quality of life at posttest (T1)*

*

*

**Figure A.6.3**

*Funnel plot of included studies on positive affect at posttest (T1)*

*

*

**Figure A.6.4**

*Funnel plot of included studies on negative affect at posttest (T1)*





**Figure A.6.5**

*Funnel plot of included studies on well-being at follow-up (T2)*

*

*

**Figure A.6.6**

*Funnel plot of included studies on positive affect at follow-up (T2)*





# Appendix A.7

Examination of potential moderators of heterogeneity, including results of meta-regression models with moderators age, gender, study region and risk of bias score

**Table A.7.1**

*Distribution of examined moderators across outcomes with significant heterogeneity*

|  | *k* | Moderator | | |  |
| --- | --- | --- | --- | --- | --- |
|  |  | Age (mean) | Female participants (%) | Study region | Risk of bias |
| Posttest (T1) | | | | |  |
| Well-being | 9 | 19.79 | 66.45 | 3:6:0 | 6:1:2 |
| Negative affect | 4 | 26.23 | 80.82 | 1:3:0 | 1:2:1 |
| Follow-Up (T2) | | | | |  |
| Well-being | 5 | 19.03 | 64.01 | 0:5:0 | 4:1:0 |
| Positive affect | 4 | 23.39 | 83.45 | 2:1:1 | 3:1:0 |

Note. k Number of primary studies; study region (1:2:3) 1 USA/Canada, 2 Europe, 3 Asia; risk of bias score (1:2:3) 1 low 2 moderate 3 high

**Table A.7.2**

Results of subgroup analysis with study region as a between-groups variable

| Tests of subgroup differences | | | | | | Effect sizes in subgroups | |
| --- | --- | --- | --- | --- | --- | --- | --- |
|  | *k* | | *Q* | *df* | *p* | | *g* (95% CI) |
| Well-being (T1) | |  |  |  |  | |  |
| 1 | | 9 | 0.20 | 1 | 0.66 | | 0.05 (-0.46 – 0.56) |
| 2 | |  |  |  |  |  | 0.12 (-0.13 – 0.36) |
| Negative affect (T1) | | | | | | | |
| 1 | | 4 | 1.94 | 1 | 0.16 | | 0.17 (-0.08 – 0.41) |
| 2 | |  |  |  |  |  | -0.15 (-0.96 – 0.66) |
| Positive affect (T2) | |  |  |  |  | |  |
| 1 | | 4 | 6.77 | 2 | 0.34 | | 0.02 (-2.23 – 2.26) |
| 2 | |  |  |  |  |  | -0.34 (-0.84 – 0.17) |
| 3 | |  |  |  |  |  | 0.55 (0.09 - 1.01) |

Note. Study region coded as 1 USA/Canada, 2 Europe, 3 Asia; k Number of primary studies; Q Q test of between-group heterogeneity; df degrees of freedom; g Hedge’s g with 95% confidence interval

**Table A.7.3**

Results of subgroup analysis with risk of bias score as a between-groups variable

| Tests of subgroup differences | | | | | | Effect sizes in subgroups | |
| --- | --- | --- | --- | --- | --- | --- | --- |
|  | *k* | | *Q* | *df* | *p* | | *g* (95% CI) |
| Well-being (T1) | |  |  |  |  | |  |
| 1 | | 9 | 0.60 | 2 | 0.74 | | 0.12 (-0.12 – 0.35) |
| 2 | |  |  |  |  |  | -0.09 (-0.60 – 0.41) |
| 3 | |  |  |  |  | | 0.12 (-2.56 – 2.80) |
| Negative affect (T1) | | | | | | | |
| 1 | | 4 | 2.88 | 2 | 0.24 | | 0.17 (-0.08 – 0.41) |
| 2 | |  |  |  |  |  | -0.09 (-4.32 – 4.14) |
| 3 | |  |  |  |  | | -0.20 (-0.56 – 0.15) |
| Positive affect (T2) | |  |  |  |  | |  |
| 1 | | 4 | 2.53 | 1 | 0.11 | | 0.18 (-0.67 – 1.03) |
| 2 | |  |  |  |  |  | -0.34 (-0.84 – 0.17) |

Note. Study region coded as 1 USA/Canada, 2 Europe, 3 Asia; k Number of primary studies; Q Q test of between-group heterogeneity; df degrees of freedom; g Hedge’s g with 95% confidence interval

**Table A.7.4**

Results of the meta-regression models with gender and age as moderators

|  | *k* | *n* | *QM* | *p* | *b_1_* | *t* |
| --- | --- | --- | --- | --- | --- | --- |
| Well-being (T1) | 9 | 2619 |  |  |  |  |
| Gender | 9 | 2619 | 0.16 | 0.70 | -0.00 | -0.39 |
| Age | 8 | 2579 | 0.24 | 0.64 | 0.01 | 0.49 |
| Negative affect (T1) | 4 | 615 |  |  |  |  |
| Gender | 4 | 615 | 0.06 | 0.84 | -0.01 | -0.24 |
| Age | 4 | 615 | 0.00 | 0.99 | 0.00 | 0.02 |
| Well-being (T2) | 5 | 2267 |  |  |  |  |
| Gender | 5 | 2267 | 1.07 | 0.38 | 0.01 | 1.03 |
| Age | 5 | 2267 | 0.50 | 0.53 | 0.01 | 0.70 |
| Positive affect (T2) | 4 | 491 |  |  |  |  |
| Gender | 4 | 491 | 1.81 | 0.31 | 0.02 | 1.35 |
| Age | 4 | 491 | 0.03 | 0.88 | -0.01 | -0.17 |

Note. k Number of primary studies; n number of participants; QM regression coefficient for the moderator; b_1_ regression coefficient for the random effect metaregression; t t value of the random slope
